# A Novel Thermal Tactile Sensor Based on Micro Thermoelectric Generator for Underwater Flow Direction Perception

**DOI:** 10.3390/s23125375

**Published:** 2023-06-06

**Authors:** Changxin Liu, Nanxi Chen, Guangyi Xing, Runhe Chen, Tong Shao, Baichuan Shan, Yilin Pan, Minyi Xu

**Affiliations:** 1 Dalian Key Lab of Marine Micro/Nano Energy and Self-Powered Systems, Marine Engineering College, Dalian Maritime University, Dalian 116026, China; 2 Artificial Intelligence College, Dalian Maritime University, Dalian 116026, China

**Keywords:** underwater perception, thermal tactile, micro thermoelectric generator, underwater flow direction identification, theoretical model

## Abstract

Underwater vehicles can operate independently in the exploitation of marine resources. However, water flow disturbance is one of the challenges underwater vehicles must face. The underwater flow direction sensing method is a feasible way to overcome the challenges but faces difficulties such as integrating the existing sensors with underwater vehicles and high-cost maintenance fees. In this research, an underwater flow direction sensing method based on the thermal tactility of the micro thermoelectric generator (MTEG) is proposed, with the theoretical model established. To verify the model, a flow direction sensing prototype is fabricated to carry out experiments under three typical working conditions. The three typical flow direction conditions are: condition No. 1, in which the flow direction is parallel to the x-axis; condition No. 2, in which the flow direction is at an angle of 45° to the x-axis; and condition No. 3, which is a variable flow direction condition based on condition No. 1 and condition No. 2. According to the experimental data, the variations and orders of the prototype output voltages under three conditions fit the theoretical model, which means the prototype can identify the flow direction of three conditions. Besides, experimental data show that in the flow velocity range of 0~5 m/s and the flow direction variation range of 0~90°, the prototype can accurately identify the flow direction in 0~2 s. The first time utilizing MTEG on underwater flow direction perception, the underwater flow direction sensing method proposed in this research is cheaper and easier to be applied on the underwater vehicles than traditional underwater flow direction sensing methods, which means it has great application prospects in underwater vehicles. Besides, the MTEG can utilize the waste heat of the underwater vehicle battery as the energy source to achieve self-powered work, which greatly enhances its practical value.

## 1. Introduction

The Remote Operated Vehicle (ROV) and Autonomous Underwater Vehicle (AUV) are underwater vehicles often utilized in underwater operations such as seabed topography surveys, underwater rescue and salvage, and marine pasture farming [1]. The performance of ROV and AUV is affected by many factors. For example, the cable affects the movement distance of ROV [2], battery power affects the endurance time of AUV, and data transmission affects the work efficiency of the AUV [3]. However, water flow disturbance, which includes the impact of high-velocity water flow and the disturbance of a vortex, is a challenge ROV and AUV both face [4].

The underwater environment is out of the ordinary. Besides high water pressure and low visibility, unpredictable strong current disturbances may lead to ROV and AUV losing balance and orientation. For ROV, losing balance may cause the cables to wrap around the ROV, which reduces the movement distance [5]. For AUV, losing balance causes the AUV to spend more power on adjusting its attitude, which further reduces the endurance time [6]. Besides, for ROV and AUV, the loss of orientation and balance not only makes it difficult to accomplish underwater tasks but also causes them to be swept away by the flow, which is a huge economic loss [7]. The underwater flow direction sensor can either be directly integrated into the underwater vehicles or installed on the manipulators of underwater vehicles. The flow direction data provided by the underwater flow direction sensor enables the underwater vehicle to quickly adapt to complex water flow disturbance when performing control algorithms [8]. Therefore, the underwater vehicles can maintain a stable attitude under the water flow disturbance, and the work efficiency of the underwater vehicles can be improved.

In addition, as one of the main tasks of underwater vehicles, regional exploration requires flow direction data to support its path planning [9,10]. Course-changing can affect the direction of the underwater vehicle. When the flow direction and the underwater vehicle direction are at a certain angle, the underwater vehicle cannot be controlled. Therefore, flow direction is an important factor that must be considered in planning the detection path of the underwater vehicle. Underwater vehicle path detection can be categorized into two methods, which are the “detection during recognition” method and the “detection before recognition” method. Based on the principle of optimal element decomposition in the coordinate search algorithm, the detection area of the “detection during recognition” method can be divided according to the flow direction in real time [9,11]. The “detection before recognition” method completes the global path planning of underwater vehicles through the Traveling Salesman Problem (TSP) shortest path algorithm [10,12]. In summary, flow direction data are an important factor affecting the attitude stability and the detection execution efficiency of the underwater vehicle.

Sensors employed to identify the velocity and direction of water flow are current meters, which can be divided into three types: mechanical current meter [13], electromagnetic current meter [14], and acoustic current meter [15].

The mechanical current meter is the earliest and most mature type with simple structure and low cost. However, the accuracy of mechanical current meter is affected by the mechanical inertia of rotor and propeller, which makes it difficult for the mechanical current meter to identify the rapidly changing water flow.

The electromagnetic current meter has flexible sampling methods and works well on surface currents affected by wind and waves. However, the accuracy of the electromagnetic current meter can be reduced once the magnetic field density is too strong or too weak, which means it cannot be utilized in some areas.

The most typical acoustic current meter is the Acoustic Doppler Current Profiler (ADCP) [16], which can be easily utilized in water and has little effect on the flow field. Besides, the ADCP can identify a three-dimensional flow. As for the Acoustic Doppler Velocity Meter (ADV) developed in recent years, it has the advantage of having little effect on the flow field and high precision [17]. The ADCP and ADV are based on the Doppler effect, while the ultrasonic time difference current meter is based on sound wave propagation. The ultrasonic time difference current meter has high accuracy and overcomes the disadvantages of the Doppler current meter due to the limitation of scattering media [18]. However, the effect of these acoustic current meters is limited by factors such as hydroacoustic, signal processing, supporting facilities, and so on. Affected by these factors, a large gap exists between the best and worst accuracy of the acoustic current meters. Furthermore, because the principle of the acoustic current meter is the propagation and the frequency shift of sound in water, the temperature of the seawater, the salt content and the size of suspended particles all have influence on the accuracy, which means it is difficult to maintain the accuracy of the current meter.

So far, the current meters have been well developed. However, there are two major challenges that the current meters face in applying to underwater vehicles. The first challenge is the cost. Due to the small size of underwater vehicles, there is little space left for current meters, which makes the installation of current meters difficult. Some current meters are expensive, causing a high installation cost. Another challenge is adaptability. The assumed carrier of the current meters is not the underwater vehicle. Therefore, the consumed power of the current meters may be a heavy burden for underwater vehicles, which may lead the performance of underwater vehicles to degrade.

Recently, ADV and ADCP have been utilized in the estimation of ocean turbulence [19] and observation of ocean current structure [20], which means ADV and ADCP have made outstanding contributions to the exploration of the ocean. However, the ADV and ADCP are also difficult to be applied to underwater vehicles. As for the mechanical current meter and electromagnetic current meter, their research has almost stopped in recent years owing to their disadvantages.

Traditional current meters generally have problems such as high cost, high energy consumption, and high difficulty in integrating into underwater vehicles, which need a long time to be solved. Due to the previous research of our team on the micro thermoelectric generator (MTEG) [21,22,23,24], we intend to propose an underwater flow direction sensing method based on MTEG to solve those problems. During this process, the microelectron mechanical systems (MEMS) thermal flow sensor has inspired us. Compared with traditional flow sensors, MEMS thermal flow sensors are manufactured by microelectronics and micromachining technology and have the advantages of small size, light weight, low cost, low power consumption, high reliability, being suitable for mass production, and ease of integration [25]. At the same time, the processing of the micron level makes it possible for MEMS thermal flow sensors to perform some special functions which traditional flow sensors cannot achieve. The MEMS thermal flow sensor identifies the flow velocity by monitoring the heat transfer, which has the advantages of high sensitivity, high accuracy, and small output signal drift [26]. Though the MEMS thermal flow sensor has many merits, it cannot be directly applied in underwater scenarios. The main reason is that the sophisticated circuitry of the MEMS thermal flow sensor can be easily corroded by water, even after some waterproof treatments. Once the circuitry is corroded, the MEMS thermal flow sensor is almost impossible to work normally. Based on the MEMS thermal flow sensor, the thermal tactile underwater flow sensing sensor proposed in this paper has the advantages of small size, light weight, low cost and ease of integration compared with traditional current meters. Besides, compared with the MEMS thermal flow sensor, the circuitry of the thermal tactile underwater flow sensing sensor does not directly contact with water, which means the circuitry is less likely to be corroded by water. Most importantly, due to the thermoelectric effect of MTEG, the thermal tactile underwater flow sensing sensor can realize self-powered work, which greatly increases its application value.

## 2. Theoretical Model

**Figure 1:**
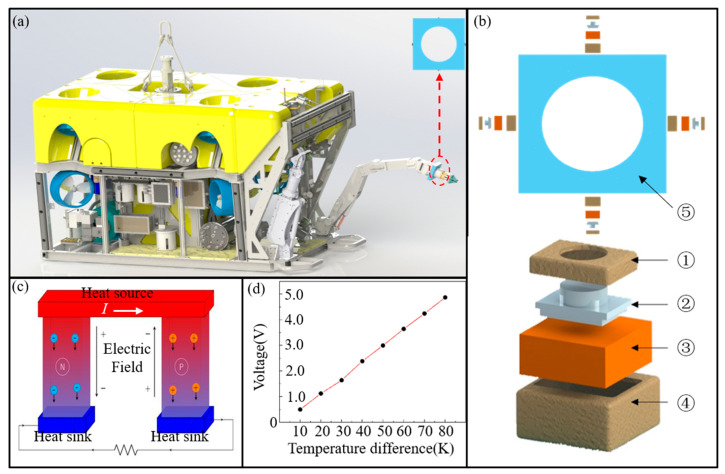
Underwater flow direction prototype based on thermal tactility of MTEG. (**a**) The flow direction sensing prototype installed in a manipulator (**b**) Schematic diagram of the flow direction sensing prototype, ① thermal tactile unit insulation layer, ② thermal tactile unit, ③ heat source, ④ heat source insulation layer, ⑤ prototype shell (**c**) schematic diagram of Seebeck effect. (**d**) V-ΔT diagram of thermal tactile unit.

Figure 1a shows the underwater flow direction prototype installed in a manipulator, and the structure of the flow direction prototype is shown in Figure 1b. The prototype consists of the thermal tactile unit insulation layer, the thermal tactile unit, the heat source, the heat source insulation layer, and the prototype shell. Among them, the function of the thermal tactile unit insulation layer and the heat source insulation layer is to keep the thermal tactile unit and the heat source insulated from the shell. The thermal tactile unit, which is the core of the prototype, converts the temperature difference between the cold end, which is close to the thermal tactile unit insulation layer, and the hot end, which is close to the heat source, into the prototype output voltage. The working principle of the thermal tactile unit is the Seebeck effect, which is shown in Figure 1c. Both ends of the P-type and N-type semiconductors are in contact with a high temperature, corresponding to the hot end, and a low temperature, corresponding to the cold end, respectively. Due to the temperature difference between the hot and cold ends, the holes in the P-type semiconductor and the electrons in the N-type semiconductor move from the hot end to the cold end. Therefore, a voltage is generated between the two ends, and the voltage is proportional to the temperature difference. The thermoelectric materials utilized in the thermal tactile unit were fabricated by our team [21], and the output voltage of the thermal tactile unit increases 0.072 V with a 1 K temperature increase, as shown in Figure 1d. The function of the heat source is to keep the hot end of the thermal tactile unit at a stable temperature. In application, the parts with high heat dissipation, such as the battery of underwater vehicles, can be utilized as the heat source.

Due to the insulating effect of the insulation layers, only the cold end of the thermal tactile unit can exchange heat with the prototype shell. Besides, since the cold end partially protrudes from the prototype shell, the cold end can exchange heat with the water, which is the premise that the prototype can identify the flow direction.

**Figure 2:**
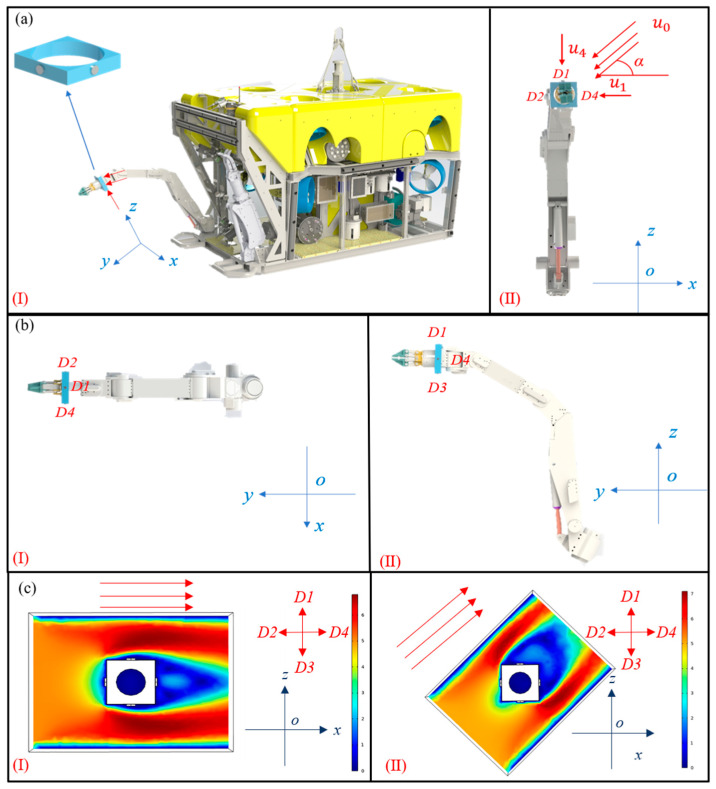
Theoretical model of underwater flow direction sensing method based on thermal tactility of MTEG. (**a**) Three-dimensional coordinate system *xyz* with manipulator as reference and top view of *xoz* planes of *xyz*. (**b**) Top view of xoy and yoz planes of *xyz*. (**c**) Simulation diagram of velocity distribution after flow direction prototype is impacted by 5 m/s water flow.

As shown in Figure 2a, a three-dimensional coordinate system xyz is established and the flow direction sensing method can identify any flow direction of the water in the xyz. The flow direction sensor can collect flow velocity data and accurately identify the flow direction. When performing control algorithms, the flow direction and velocity data can make the underwater vehicle or manipulator better cope with the complex underwater environment. Therefore, the difficulty of stabilizing the attitude of the underwater vehicle or manipulator is reduced, and the efficiency and safety of the underwater vehicle when performing the area detection task are improved.

The manipulator is the main tool of the underwater vehicle when performing tasks, but the small volume and low weight of the manipulator make it more difficult to deal with the water flow disturbance. At the same time, the volume of the manipulator also makes it more difficult to apply the traditional current meters, which means it needs a new current meter more urgently than the underwater vehicle. Therefore, the research selects the manipulator to verify the feasibility of the flow direction sensing method based on the thermal tactility of MTEG. For the manipulator, the most important factor in the attitude stability and motion of the manipulator is the vertical water flow, as shown by the red arrows in Figure 2a. Therefore, the vertical flow of the manipulator is selected to verify the flow direction sensing method. Thus, the coordinate system xoz is established on the plane where the four thermal tactile sensors D1, D2, D3, and D4 are located, as shown in Figure 2b. The x-axis is parallel to the line between D2 and D4, and the x-axis is parallel to the line between D1 and D3.

The core of the thermal tactile sensor is MTEG. According to the principle of MTEG, the faster the water flow velocity, the greater the temperature difference between the cold end and the hot end of MTEG. Finally, the output voltage of MTEG increases. The relationship between the output voltage of MTEG and the water flow velocity is derived in detail in the Supporting Information, and here, we only give the relationship:

(1) $$V=k_{1}+k_{2}u^{\frac{1}{2}}$$

V is the output voltage of METG, $k_{1}$ and $k_{2}$ are constants defined by the characteristics of MTEG, and u is the flow velocity of the water which flows through MTEG. Generally, the flow velocity of the water which flows through MTEG is the flow velocity of the water which flows through the thermal tactile sensor.

The realization of flow direction perception is based on Equation (1).

The operating conditions analyzed in this paper are shown in Figure 2a(II), in which water flows at a speed $u_{0}$ through a plane composed of four sensor devices at an angle α to the x-axis. The analysis of water flow through a plate is one of the research hotspots in the field of hydrodynamics, which usually includes the analysis of flow pressure, flow velocity, flow rate, and other parameters. Some researchers choose to simplify the flow field by the vector decomposition method in the flow velocity analysis so that they can focus on the analysis and calculation of the flow velocity [27,28,29]. Since the water flow velocity directly affects the output performance of MTEG, the flow velocity sensing is an important factor affecting the conclusion of the flow direction sensing method proposed in this paper. Therefore, based on the relevant research conclusions, the vector decomposition method is also adopted to simplify the flow field analysis in this research.

Ideally, the water flow can be decomposed into $u_{1}$, which is parallel to the x-axis, and $u_{4}$, which is parallel to the x-axis. The $u_{1}$ flows through D1, and the $u_{4}$ flows through D4. The velocity of the $u_{1}$ and $u_{4}$ can be calculated by the following equations:

(2) $$u_{1}=u_{0}cos\alpha$$

(3) $$u_{4}=u_{0}sin\alpha$$

Therefore, α can be calculated by the following equation:

(4) $$\alpha =\tan^{-1}\left(u_{4}/u_{1}\right)$$

Combining Equation (1) and Equation (4), α can also be calculated by the following equation:

(5) $$\alpha =\tan^{-1}\left[\frac{{\left(V_{D4}-k_{1}\right)}^{2}}{{\left(V_{D1}-k_{1}\right)}^{2}}\right]$$

Because D1 is symmetrical with D3, D2 is symmetrical with D4, and 90≥α≥0; the velocity of $u_{2}$, which flows through D2, is less than the velocity of $u_{4}$, and the velocity of $u_{3}$, which flows through D3, is less than the velocity of $u_{1}$. The output voltages of different thermal tactile sensors can be obtained by Equation (1). By comparing the output voltages of four thermal tactile sensors, the water flow at an angle of α can be identified.

The water flow at an angle of α can be identified similarly when 180≥α≥90, 270≥α≥180, and 360≥α≥270.

The study simulates the flow of two typical angles, as shown in Figure 2c. Figure 2c(I) shows the flow of α=180°, $u_{0}=5\;m/s$, and Figure 2c(II) shows the flow of α=225°, $u_{0}=5\;m/s$.

In Figure 2c(I), $u_{1}\approx u_{3}\approx 5\;m/s$, which is consistent with the theoretical model but different from the theoretical model, $u_{2}\neq 0,u_{4}\neq 0$. This is because the velocity of the water flow cannot be zeroed due to the existence of obstacles, so the velocity of $u_{2}$ is not the same as 0 derived from the theoretical model. However, $u_{4}\leq u_{2}$ is still consistent with the theoretical model. Therefore, $V_{D1}=V_{D3}>V_{D2}>V_{D4}$; the water flow direction can be identified by comparing the output voltages of four thermal tactile sensors.

In Figure 2c(II), $u_{2}\approx u_{3}\approx 4\;m/s$, $u_{1}\leq u_{3}$, $u_{4}\leq u_{2}$. The simulation of water flow is consistent with the derivation of the theoretical model. Therefore, $V_{D2}=V_{D3}>V_{D4}=V_{D1}$, the water flow direction can be identified by comparing the output voltages of four thermal tactile sensors.

It can be seen from Figure 2c that the simulation of water flow is consistent with the theoretical model except for rare special angles. Although the simulation is not consistent with the theoretical model at those special angles, it does not affect the perception of the flow direction. For ease of comparison, the matrices A and B are set as follows:

(6) $$A=\left[\begin{array}{cc} V_{D1} & V_{D2}\\ V_{D3} & V_{D4} \end{array}\right]$$

(7) $$B=\left[\begin{array}{cc} V_{D1} & V_{D3}\\ V_{D4} & V_{D2} \end{array}\right]$$

The results of matrices comparison and flow direction are shown in Table 1.

## 3. Results and Discussions

### 3.1. Experimental System

**Figure 3:**
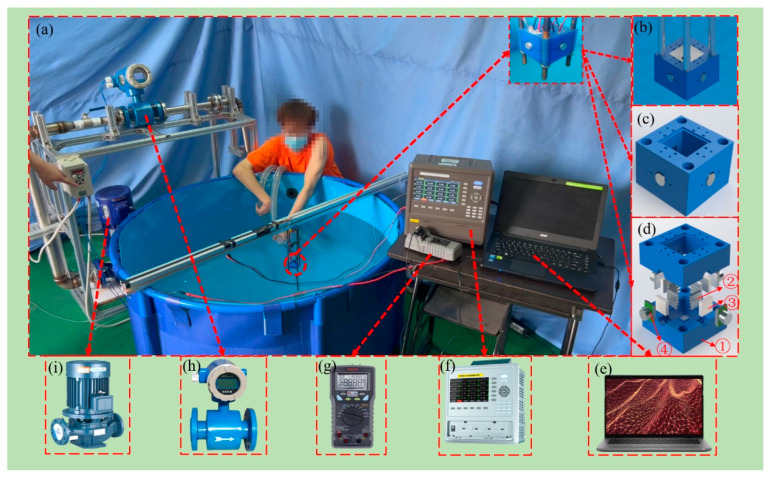
Underwater flow direction sensing experimental system. (**a**) Real photograph of experimental system, (**b**) Installation of the flow direction sensing prototype, (**c**) 3D diagram of the prototype, (**d**) Structure of the prototype, ① shell, ② heat dissipation unit, ③ temperature control unit, ④ thermal tactile unit, (**e**) Data processing terminal, (**f**) Temperature sensing unit, (**g**) Voltage acquisition unit, (**h**) Electromagnetic flowmeter, (**i**) Centrifugal pump.

In order to verify the theoretical model and test the performance of the underwater flow direction sensing prototype, a flow direction sensing experimental system is designed, as shown in Figure 3.

Figure 3a is a real photograph of the experimental system, and the installation position of the flow direction sensing prototype is shown by the red circle in the figure. The installation of the prototype is shown in Figure 3b, and the 3D diagram of the prototype is shown in Figure 3c. The prototype consists of four heat dissipation units, four temperature control units based on the Peltier effect, four thermal tactile units and a shell, as shown in Figure 3d. The heat dissipation units and the shell create an insulating environment for the thermal tactile units and the temperature control units, corresponding to ①, ④, and ⑤ in Figure 1b. The temperature control units maintain the hot end of the thermal tactile units at a constant temperature, corresponding to ③ in Figure 1b. In experiment, the input power of a temperature control unit is in the range 2~3 W. The thermal tactile units transform the heat exchange between the thermal tactile unit and the environment into voltage to identify the flow direction of the water, which corresponds to ② in Figure 1b. The dimension of the thermal tactile unit is 9 × 11 × 8 mm^3^ and the dimension of the prototype is 50 × 50 × 34 mm^3^.

The prototype is connected with the temperature sensing unit shown in Figure 3f and the voltage acquisition unit shown in Figure 3g. The temperature sensing unit is utilized to monitor the temperature of the heat source in real time. The voltage acquisition unit is utilized to collect the prototype output voltage and input it to the data processing terminal shown in Figure 3e in real time. During the experiment, the water flow velocity is adjusted by adjusting the rotational speed of the centrifugal pump shown in Figure 3i and monitored by the electromagnetic flowmeter shown in Figure 3h. The direction of the flow is manually adjusted with an elongated plastic hose to flow over the prototype. Once the flow velocity or flow direction is changed, the voltage acquisition unit records the output voltage of the prototype and input the voltage data to the data processing terminal.

Three typical flow direction conditions are set up to verify the performance of the prototype. They are condition No. 1, in which the flow direction is parallel to the x-axis, and condition No. 2, in which the flow direction is at an angle of 45° to the x-axis. As for condition No. 3, it is a variable flow direction condition based on condition No. 1 and condition No. 2. In the three conditions, the hot end temperature of the thermal tactile units is set at 313 K, which is the normal working temperature of the lithium battery pack utilized in the underwater vehicle [30]. Besides, the water temperature is 293 K, which is the surface temperature of the sea in summer [31]. Therefore, the initial temperature difference of the thermal tactile units is 20 K.

### 3.2. Discussions

#### 3.2.1. When the Flow Direction Is Parallel to the x-Axis (Condition No. 1)

**Figure 4:**
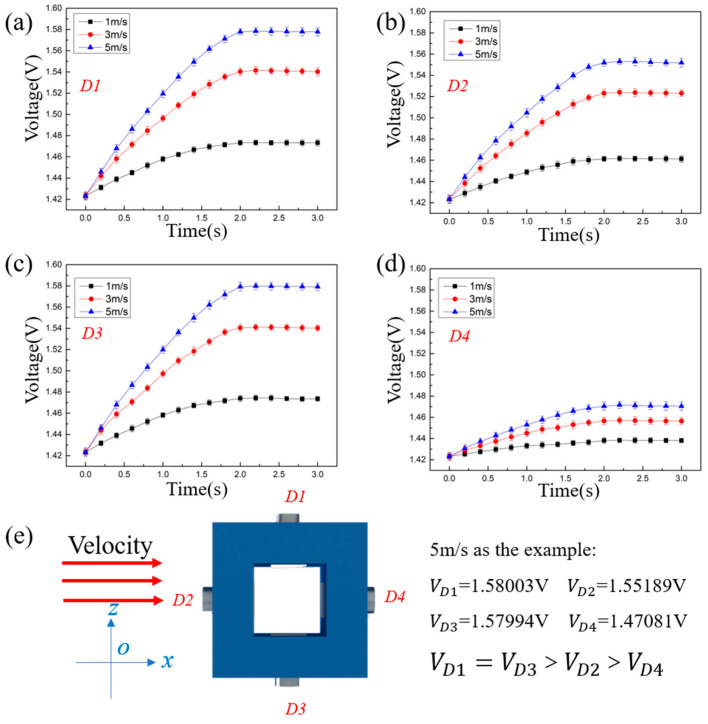
Output voltage of the prototype under condition No. 1. (**a**) Output voltage of D1 (**b**) Output voltage of D2 (**c**) Output voltage of D3 (**d**) Output voltage curve of D4 (**e**) Schematic diagram of the water flow impacts the prototype.

As shown in Figure 4e, the flow direction is controlled to be parallel to the x-axis. The velocity of the flow is set at 1 m/s, 3 m/s, and 5 m/s to verify whether the prototype can identify the flow direction at different velocities.

Figure 4 is the performance of the prototype under condition No. 1. According to the theoretical model, the order of the output voltage under the impact of water flow shown in Figure 4e is $V_{D1}=V_{D3}>V_{D2}>V_{D4}$. From Figure 4a–d, it can be seen that under the flow velocity of 1 m/s, 3 m/s, and 5 m/s, the output voltages become stable after a continuous increase of 2 s, and the output voltages are $V_{D1}\approx V_{D3}>V_{D2}>V_{D4}$. Therefore, the order of the output voltages is always $V_{D1}\approx V_{D3}>V_{D2}>V_{D4}$, whether at a flow velocity of 1 m/s, 3 m/s, or 5 m/s. The order is in accord with the theoretical model, which means that the prototype can correctly identify the flow direction under condition No. 1.

#### 3.2.2. When the Flow Direction Is at an Angle of 45° to the x-Axis (Condition No. 2)

**Figure 5:**
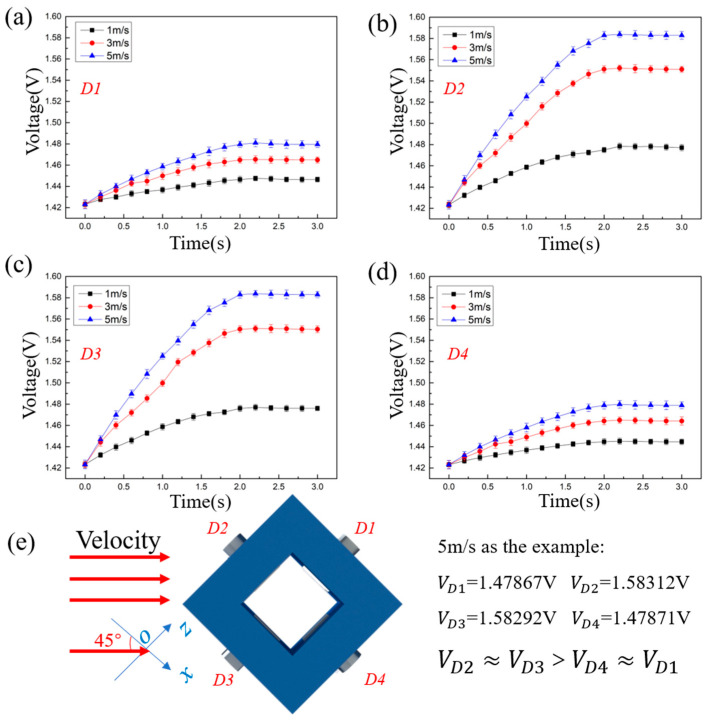
Output voltage of the prototype under condition No. 2. (**a**) Output voltage of D1. (**b**) Output voltage of D2. (**c**) Output voltage of D3. (**d**) Output voltage curve of D4. (**e**) Schematic diagram of the water flow impacts the prototype.

As shown in Figure 5e, the flow direction is controlled to be at an angle of 45° to the x-axis. The velocity of flow is set at 1 m/s, 3 m/s, and 5 m/s.

Figure 5 is the performance of the prototype under condition No. 2. According to the theoretical model, the order of the output voltage under the impact of water flow shown in Figure 5e is $V_{D2}=V_{D3}>V_{D4}=V_{D1}$. From Figure 5a–d, it can be seen that under the flow velocity of 1 m/s, 3 m/s, and 5 m/s, the output voltages become stable after a continuous increase of 2 s, and the output voltages are $V_{D2}\approx V_{D3}>V_{D4}\approx V_{D1}$. Therefore, whether at a flow velocity of 1 m/s, 3 m/s, or 5 m/s, the order of the output voltages is always $V_{D2}\approx V_{D3}>V_{D4}\approx V_{D1}$. The order is in accord with the theoretical model, which means that the prototype can still correctly identify the flow direction under condition No. 2.

#### 3.2.3. Variable Flow Direction Condition (Condition No. 3)

**Figure 6:**
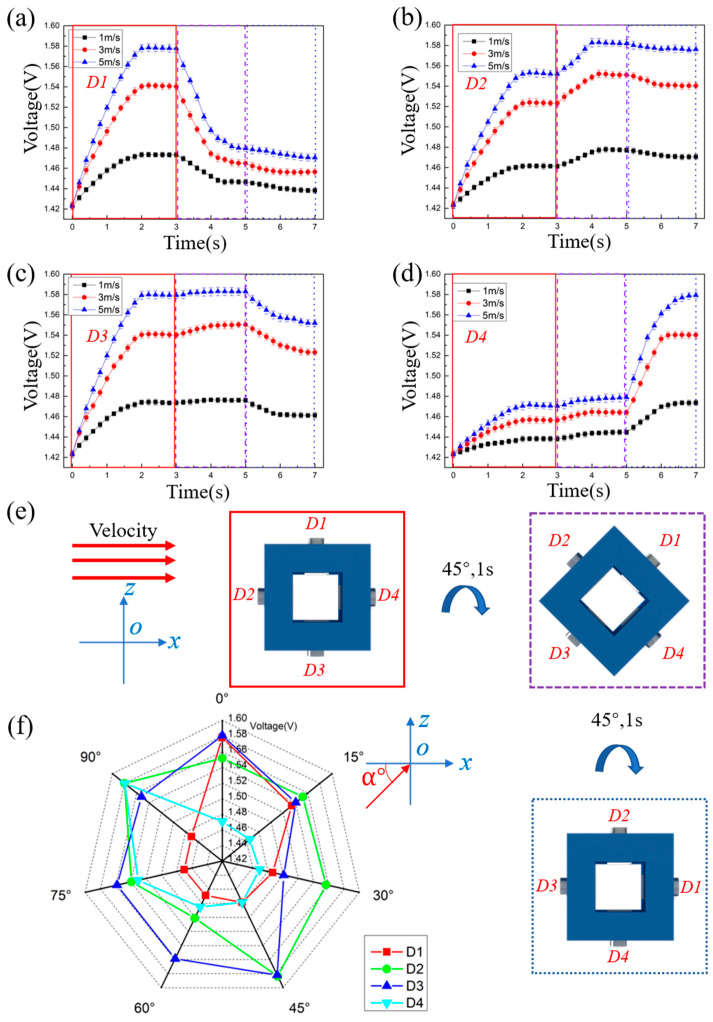
Output voltage of the prototype under condition No. 3. (**a**) Output voltage of the D1. (**b**) Output voltage of the D2. (**c**) Output voltage of the D3. (**d**) Output voltage curve of the D4. (**e**) Schematic diagram of the water flow impacts the prototype. (**f**) Output voltage of four thermal tactile sensors for flow direction measurement in a full range of 90° at 5 m/s.

As shown in Figure 6e, the flow is controlled parallel to the x-axis. In 0~3 s, the prototype keeps still. In 3~4 s, the prototype rotates 45° at a speed of 45°/s. In 4~5 s, the prototype keeps still. In 5~6 s, the prototype rotates 45° at a speed of 45°/s. In 6~7 s, the prototype keeps still. The velocity of flow is set at 1 m/s, 3 m/s, and 5 m/s.

Figure 6 is the performance of the prototype under condition No. 3. According to the theoretical model, the order of the output voltage under the impact of water flow shown in Figure 6e is $V_{D1}=V_{D3}>V_{D2}>V_{D4}$ in 0~3 s, $V_{D2}=V_{D3}>V_{D4}=V_{D1}$ in 3~5 s, and $V_{D2}=V_{D4}>V_{3}>V_{D1}$ after 5 s. From Figure 6a–d, it can be seen that under the flow velocity of 1 m/s, 3 m/s, and 5 m/s, the output voltages is $V_{D1}\approx V_{D3}>V_{D2}>V_{D4}$ in 0~3 s, $V_{D2}\approx V_{D3}>V_{D4}\approx V_{D1}$ in 3~5 s, and $V_{D2}\approx V_{D4}>V_{D3}>V_{D1}$ after 5 s. Obviously, the order is in accord with the theoretical model, which means that the prototype can correctly identify the variable flow direction under condition No. 3.

Figure 6f is the output voltage of four thermal tactile sensors for flow direction measurement in a full range of 90° at 5 m/s. As shown in Figure 6f, within the water flow angle of 0~90°, the voltage curve of D1 is similar to the voltage curve of D4, and the voltage curve of D2 is similar to the voltage curve of D3. According to Figure 6f, the voltage variations of four thermal tactile sensors are regular, which means it is feasible for the prototype to identify the water flow from 0° to 90°.

The underwater thermal tactile direction sensing prototype needs to be further optimized, but it still has some merits compared to the traditional current meters and MEMS thermal flow sensor. The comparisons are shown in the Table 2. For a more detailed comparison of data, such as price, accuracy, dimension, etc., please refer to Table S1 of the Supporting Information.

## 4. Implications and Prospects

This research studies whether the flow direction sensing prototype based on the flow direction sensing method can successfully realize the direction perception at some angles. Although the three conditions in this paper can represent any flow direction in the whole two-dimensional coordinate system, more experimental data are still necessary to support it. Therefore, in the future, we need to perform experiments on the entire two-dimensional coordinate system. In fact, we have already tested the flow direction sensing prototype when the flow direction is at an angle of 15°, 30°, 60°, 75°, 90° to the x-axis and put the data in the Supporting Information, but we know those data are far from enough. Besides, if we want to make the flow direction sensing prototype better applied in practice, we need to study whether it can realize direction perception in a three-dimensional coordinate system. In order to identify the flow direction in the three-dimensional coordinate system, the prototype and the theoretical model in this research need to be updated. In summary, there are much work to do in the future.

## 5. Conclusions

An underwater flow direction sensing method based on the thermal tactility of MTEG is proposed, and a theoretical model is established in this research. Based on the theoretical model, a prototype is fabricated to carry out experiments under three typical conditions. The method compares the voltages which are obtained by different thermal tactile sensors of the prototype to identify the flow direction. Under the three typical conditions, the variations and orders of four thermal tactile sensors voltages fit the theoretical model, indicating that the method can identify underwater flow direction. For example, when the flow direction is parallel to the x-axis, the comparison result of water flow velocity through four thermal tactile sensors is $u_{1}\approx u_{3}>u_{2}>u_{4}$ according to the theoretical model. Therefore, the comparison result of voltage should be $V_{D1}\approx V_{D3}>V_{D2}>V_{D4}$. According to Figure 4, the comparison result of the voltages of four thermal tactile sensors is indeed $V_{D1}\approx V_{D3}>V_{D2}>V_{D4}$. It can be seen that the experimental data are consistent with the theoretical model. Besides, according to Figure 4, the voltages of the four thermal tactile sensors can be clearly distinguished since the water flowed over the prototype for 1 s, and the voltages remain stable after 2 s. Therefore, the prototype can identify the flow direction parallel to the x-axis within 2 s. Thus, the prototype can achieve accurate identification in 0~2 s and is not affected by variation of the flow velocity.

Though the accuracy of the prototype is far from those traditional sensors, it is mainly because the prototype requires further circuit design and algorithm optimization. Once the prototype can be further optimized, the accuracy can be greatly improved and may be possible to get close to the traditional sensors. In fact, according to the theoretical model, the accuracy of the thermal tactile sensor can even reach 1°. Despite the accuracy, the most important merit of the prototype is that it can utilize the battery waste heat as the energy source to achieve self-powered work. Compared with traditional methods, the underwater thermal tactile direction perception proposed in this paper firstly utilizes a micro thermoelectric generator (MTEG) on underwater flow direction perception. MTEG can convert the temperature difference between the underwater vehicle battery and the water into voltage, which means the battery waste heat is converted into electricity by MTEG. Besides, the prototype has merits such as low cost, low operation difficulty, low maintenance difficulty, and so on. Therefore, it not only greatly reduces difficulty and cost in the application of underwater vehicles, but also greatly promotes the application of underwater flow direction sensors in underwater vehicles.

## Figures and Tables

**Table 1:** The matrices comparison and flow direction.

$\mathrm{Comparison}\;\mathrm{of}\;\left|A\right|$ $\;\mathrm{and}\;\left|B\right|$	α
$\left|A\right|<0,\left|A\right|/\left|B\right|\approx -1$	α=0
$\left|A\right|>0,\left|\left|A\right|/\left|B\right|\right|>1$	90>α>0 $\alpha =\tan^{-1}\left[\frac{{\left(V_{D4}-k_{1}\right)}^{2}}{{\left(V_{D1}-k_{1}\right)}^{2}}\right]$
$\left|A\right|<0,\left|A\right|/\left|B\right|\approx 1$	α=90
$\left|B\right|<0,\left|\left|A\right|/\left|B\right|\right|<1$	180>α>90 $\alpha =\tan^{-1}\left[-\frac{{\left(V_{D2}-k_{1}\right)}^{2}}{{\left(V_{D1}-k_{1}\right)}^{2}}\right]$
$\left|A\right|>0,\left|A\right|/\left|B\right|\approx -1$	α=180
$\left|A\right|\langle 0,\left|\left|A\right|/\left|B\right|\right|\rangle 1$	270>α>180 $\alpha =\tan^{-1}\left[\frac{{\left(V_{D2}-k_{1}\right)}^{2}}{{\left(V_{D3}-k_{1}\right)}^{2}}\right]$
$\left|A\right|>0,\left|A\right|/\left|B\right|\approx 1$	α=270
$\left|B\right|>0,\left|\left|A\right|/\left|B\right|\right|<1$	360>α>270 $\alpha =\tan^{-1}\left[-\frac{{\left(V_{D4}-k_{1}\right)}^{2}}{{\left(V_{D3}-k_{1}\right)}^{2}}\right]$

**Table 2:** The comparisons between traditional sensors and ours.

Sensor Type	Paragraph	Working Principle	Merits	Defects
Mechanical Current Meter	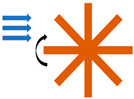	Mechanical transmission	Easy to be manufactured and maintained	Easy to be influenced by water flow disturbance
Electromagnetic Current Meter	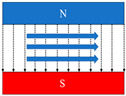	Faraday law of electromagnetic induction	Long service life, variable sampling method	Vulnerable to electromagnetic interference
Acoustic Current Meter	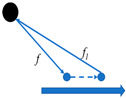	Doppler principle	High accuracy, wide measuring range	Hard to be maintained
MEMS Thermal Flow Senor	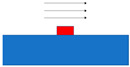	Heat conduction and heat convection	High accuracy, small volume, easy to be installed	Hard to be applied underwater
Thermal Tactile Underwater Flow Direction Sensor	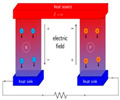	Heat conduction, heat convection, and thermoelectric effect	Cheap, small volume, easy to be installed and maintained	Low accuracy, narrow measuring range

## Data Availability

Not applicable.

## Supplementary Materials

The following are available online at https://www.mdpi.com/article/10.3390/s23125375/s1, Figure S1. Thermodynamic model of the flow velocity prototype. Figure S2. The marks of the water flow angle. Figure S3. Output voltage of the prototype when the flow direction is 15° to the x-axis. Figure S4. Output voltage of the prototype when the flow direction is 30° to the x-axis. Figure S5. Output voltage of the prototype when the flow direction is 60° to the x-axis. Figure S6. Output voltage of the prototype when the flow direction is 75° to the x-axis. Figure S7. Output voltage of the prototype when the flow direction is 90° to the x-axis. Table S1. The performance comparisons between traditional sensors and the thermal tactile sensor. Video S1. Flow direction experiment video when the flow direction is parallel to the x-axis. Video S2. Flow direction experiment video when the flow direction is at an angle of 45° to the x-axis. References [23,24,32,33] are cited in the Supplementary Materials.

## Nomenclature

List of acronyms and symbols:

Acronyms and symbols	Representative meaning	Unit
MTEG	Micro Thermoelectric Generator	/
ROV	Remote Operated Vehicle	/
AUV	Autonomous Underwater Vehicle	/
ADCP	Acoustic Doppler Current Profiler	/
ADV	Acoustic Doppler Velocity Meter	/
TSP	Traveling Salesman Problem	/
MEMS	Microelectron Mechanical Systems	/
D1	Thermal tactile sensor No. 1	/
D2	Thermal tactile sensor No. 2	/
D3	Thermal tactile sensor No. 3	/
D4	Thermal tactile sensor No. 4	/
V	Output voltage of MTEG	V
u	Flow velocity	m/s
$k_{1}$	A constant defined by the characteristics of MTEG	V
$k_{2}$	A constant defined by the characteristics of MTEG	V/(m/s)
$u_{1}$	Velocity of the water flow over D1	m/s
$u_{2}$	Velocity of the water flow over D2	m/s
$u_{3}$	Velocity of the water flow over D3	m/s
$u_{4}$	Velocity of the water flow over D4	m/s

## References

[1] Gao D., Wang T., Qin F., Zhang S., Jing J., Yang J. (2022). Design, fabrication, and testing of a maneuverable underwater vehicle with a hybrid propulsor. Biomim. Intell. Robot..

[2] Long C., Hu M., Qin X., Bian Y. (2022). Hierarchical trajectory tracking control for ROVs subject to disturbances and parametric uncertainties. Ocean. Eng..

[3] Zhang Z., Lin M., Li D. (2022). A double-loop control framework for AUV trajectory tracking under model parameters uncertainties and time-varying currents. Ocean Eng..

[4] Boyuan Y., Chu V.H. (2022). The front runner in roll waves produced by local disturbances. J. Fluid Mech..

[5] Zin H.T., Hiroyoshi S., Daniel G.-V. (2022). On the theory and application of absolute coordinates-based multibody modelling of the rigid–flexible coupled dynamics of a deep-sea ROV-TMS (tether management system) integrated model. Ocean Eng..

[6] Mohsan S.A.H., Khan M.A., Mazinani A., Alsharif M.H., Cho H.S. (2022). Enabling Underwater Wireless Power Transfer towards Sixth Generation (6G) Wireless Networks: Opportunities, Recent Advances, and Technical Challenges. J. Mar. Sci. Eng..

[7] Wu L., Li S., Feng X., Jiang H., Zhang X., Hu W. (2022). Unsteady simulation of AUVs approaching seafloor by self-propulsion using multi-block hybrid dynamic grid method. J. Fluids Struct..

[8] Lin Y.H., Siddall R., Schwab F., Fukushima T., Banerjee H., Baek Y., Vogt D., Park Y.-L., Jusufi A. (2021). Modeling and Control of a Soft Robotic Fish with Integrated Soft Sensing. Adv. Intell. Syst..

[9] Lv T., Zhang M., Wang Y. (2022). Prediction-Based Region Tracking Control Scheme for Autonomous Underwater Vehicle. J. Mar. Sci. Eng..

[10] Chu Z., Chen Y., Zhu D., Zhang M. (2021). Observer-based adaptive neural sliding mode trajectory tracking control for remotely operated vehicles with thruster constraints. Trans. Inst. Meas. Control.

[11] Liu X., Zhang M., Yao F., Yin B., Chen J. (2021). Barrier Lyapunov function based adaptive region tracking control for underwater vehicles with thruster saturation and dead zone. J. Frankl. Inst..

[12] Li C., Huang X., Ding J., Song K., Lu S. (2022). Global path planning based on a bidirectional alternating search A* algorithm for mobile robots. Comput. Ind. Eng..

[13] Loder J.W., Hamilton J.M. (1991). Degradation of some mechanical current meter measurements by high-frequency mooring or wave motion. IEEE J. Ocean. Eng. A J. Devoted Appl. Electr. Electron. Eng. Ocean. Environ..

[14] MacVicar B.J., Beaulieu E., Champagne V., Roy A.G. (2007). Measuring water velocity in highly turbulent flows: Field tests of an electromagnetic current meter (ECM) and an acoustic Doppler velocimeter (ADV). Earth Surf. Process. Landf..

[15] Llaban A.B., Ella V.B. (2022). Conventional and sensor-based streamflow data acquisition system for sustainable water resources management and agricultural applications: An extensive review of literature. IOP Conf. Ser. Earth Environ. Sci..

[16] Dunn M., Zedel L. (2022). Evaluation of discrete target detection with an acoustic Doppler current profiler. Limnol. Oceanogr. Methods.

[17] Kim Y., Oh S., Lee S., Byun J., An H. (2021). Application of Stage-Fall-Discharge Rating Curves to a Reservoir Based on Acoustic Doppler Velocity Meter Measurement Data. Water.

[18] Xingyi H.U. (2019). Application of Venturi Method in Calculation of River Flow. J. China Hydrol..

[19] Bae H.S., Kim W.K., Son S.U., Kim W.S., Park J.S. (2022). An Estimation of the Backscattering Strength of Artificial Bubbles Using an Acoustic Doppler Current Profiler. Sensors.

[20] Goswami A. (2021). Acoustic Doppler Current Profiler to Measure Current Velocity. J. Oceanogr. Mar. Res..

[21] Liu C., Zhao K., Fan Y., Gao Y., Zhou Z., Li M., Gao Y., Han Z., Xu M., Pan X. (2022). A flexible thermoelectric film based on Bi2Te3 for wearable applications. Funct. Mater. Lett..

[22] Liu C.X., Ye W.X., Li H.A., Liu J.H., Zhao C., Mao Z.F., Pan X.X. (2021). Experimental study on cascade utilization of ship’s waste heat based on TEG-ORC combined cycle. Int. J. Energy Res..

[23] Liu C., Shan B., Chen N., Liu J., Zhou Z., Wang Q., Gao Y., Han Z., Liu Z., Xu M. (2022). A material recognition method for underwater application based on Micro Thermoelectric Generator. Sens. Actuators A Phys..

[24] Liu C., Qu G., Shan B., Aranda R., Chen N., Li H., Zhou Z., Yu T., Wang C., Mi J. (2023). Underwater Hybrid Energy Harvesting based on TENG-MTEG for Self-powered Marine Mammal Condition Monitoring System. Mater. Today Sustain..

[25] Ejeian F., Azadi S., Razmjou A., Orooji Y., Kottapalli A., Warkiani M.E., Asadnia M. (2019). Design and applications of MEMS flow sensors: A review. Sens. Actuators A Phys..

[26] Silvestri S., Schena E. (2012). Micromachined flow sensors in biomedical applications. Micromachines.

[27] Ye C.J., Huang H.L., Rao X., Chen S. (2015). Analysis of the flow properties on a moving flat plate impinged by an inclined water jet flow. Chin. J. Hydrodyn..

[28] Wang C., Wang X., Shi W., Lu W., Tan S.K., Zhou L. (2017). Experimental investigation on impingement of a submerged circular water jet at varying impinging angles and Reynolds numbers. Exp. Therm. Fluid Sci..

[29] Chen X.X., Wang C., Shi W.D., Zhang Y.C. (2020). Numerical simulation of submerged impinging water jet at different impact angles. J. Drain. Irrig. Mach. Eng. JDIME.

[30] Ma S., Jiang M., Tao P., Song C., Wu J., Wang J., Deng T., Shang W. (2018). Temperature effect and thermal impact in lithium-ion batteries: A review. Prog. Nat. Sci. Mater. Int..

[31] Nonaka M., Xie S.P. (2003). Covariations of Sea Surface Temperature and Wind over the Kuroshio and Its Extension: Evidence for Ocean-to-Atmosphere Feedback. J. Clim..

[32] Li G., Zhu Z., Zheng Y., Guo W., Tang Y. (2022). Development of a powerful hybrid micro thermoelectric generator based on an ultrahigh capacity miniature combustor. Appl. Therm. Eng..

[33] Tao W.Q. (2019). Heat Transfer.

