# Supplementary material for: A Novel Thermal Tactile Sensor Based on Micro Thermoelectric Generator for Underwater Flow Direction Perception
Source: Sensors (Basel). 2023 Jun 6;23(12):5375. doi: 10.3390/s23125375 (PMC10302903; doi:10.3390/s23125375)
Supplement: Supplementary file 1 [file sensors-23-05375-s001.zip › sensors-2387004-supplementary.pdf]

A Novel Thermal Tactile Sensor Based on Micro Thermoelectric Generator for Underwater Flow Direction Perception

Changxin Liu [1,*,†], Nanxi Chen [1,†], Guangyi Xing [1], Runhe Chen [1], Tong Shao [1], Baichan Shan [1], Yilin Pan [2] and Minyi Xu [1,*]

[1] Dalian Key Lab of Marine Micro/Nano Energy and Self-Powered Systems, Marine Engineering College, Dalian Maritime University, Dalian 116026, China
[2] Artificial Intelligence College, Dalian Maritime University, Dalian 116026, China
* Correspondence: liu_changxin@dlmu.edu.cn (C.L.); xuminyi@dlmu.edu.cn (M.X.)
† These authors contributed equally to this work.

Files include:

Supporting Note S1: Derivation of relationship between flow velocity and Micro Thermoelectric Generator (MTEG) output voltage.

Figure S1. Thermodynamic model of the flow velocity prototype.

Figure S2. The marks of water flow angle.

Figure S3. Output voltage of the prototype when the flow direction is 15° to the x-axis.

Figure S4. Output voltage of the prototype when the flow direction is 30° to the x-axis.

Figure S5. Output voltage of the prototype when the flow direction is 60° to the x-axis.

Figure S6. Output voltage of the prototype when the flow direction is 75° to the x-axis.

Figure S7. Output voltage of the prototype when the flow direction is 90° to the x-axis.

Table S1. The performance comparisons between traditional sensors and thermal tactile sensor.

Video S1. Flow direction experiment video when the flow direction is parallel to the x-axis.

Video S2. Flow direction experiment video when the flow direction is at an angle of 45° to the x-axis.

Supporting Note S1

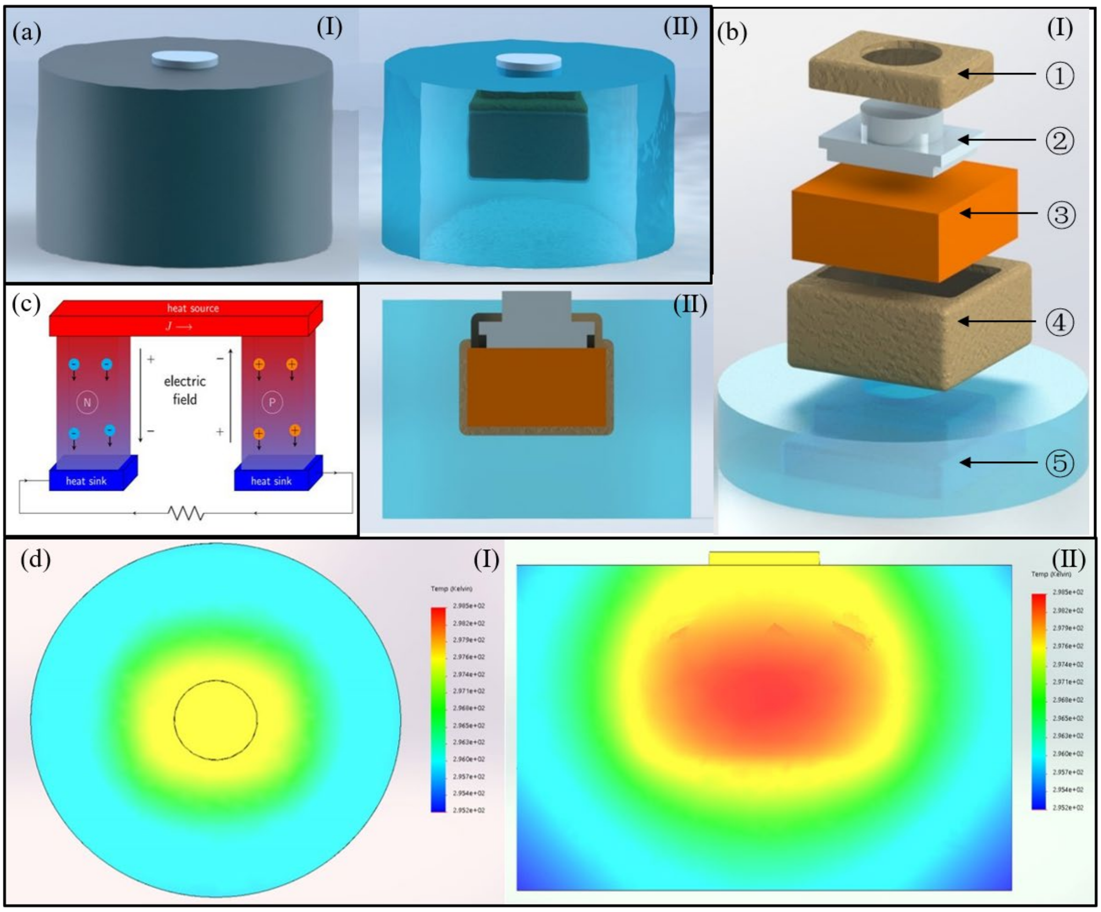

Figure S1. Thermodynamic model of the flow velocity sensing theory (a)Schematic diagram of the flow velocity prototype (b)Schematic diagram of the structure of the flow velocity prototype, ①the insulation layer of TTU, ②the thermal tactile unit(TTU), ③the heat source, ④the insulation layer of heat source, ⑤the prototype shell (c)Seebeck effect (d)Heat transfer simulation of the flow velocity prototype

Fig.S1(a) is the schematic diagram of the flow velocity sensing prototype and the structure of the prototype is shown in Fig.S1(b). The prototype can be divided into five parts, which are the insulation layer of thermal tactile unit (TTU), the TTU, the heat source, the insulation layer of heat source, and the prototype shell. Among them, the function of two insulation layers is to insulate the TTU and the heat source from the prototype shell. The MTEG is the core part of TTU and its working principle, the Seebeck effect, is shown in Fig.S1(c) [26]. Both semiconductor of P-type and semiconductor of N-type are in contact with high temperature and low temperature, the end which is in contact with the high temperature is the hot end, and the end which is in contact with the low temperature is the cold end. For the TTU, the cold end is connected to the insulation layer of TTU, and the hot end is connected to the heat source. Due to the temperature difference of the two ends, the holes of the P-type semiconductor and the electrons of the N-type semiconductor move from the hot end to the cold end. With the accumulation of the holes and electrons, the voltage is generated and it increases with the temperature difference increasing [2]. Lastly, the heat source is utilized to keep the hot end of the TTU at a stable temperature.

As shown in Fig.S1(d)(I), due to the insulation effect of two insulation layers, the heat transfer between the prototype and the water is Φ_{cond} while the water is resting. Φ_{cond} is the conductive heat transfer between the water and the cold end of the TTU. While the water flows, the heat transfer between the prototype and the water are Φ_{cond} and Φ_{conv}. Φ_{conv} is the convective heat transfer between the water and the cold end of the TTU.

The equation of conductive heat transfer Φ_{cond} is as follows [3]:

$$\Phi_{cond} = \lambda_w A(T_{Co} - T_w)/\delta D \quad \text{(S1)}$$

T_{Co} is the cold end temperature of the TTU, T_w is the water temperature, A is the contact area between the TTU and the water, δD is the thickness of the thermal conductive layer between the TTU and the water, and λ_w is the thermal conductivity of the water.

In the broad water, due to the fluidity, mass and thermal conductivity of the water, the heat transferred from the cold end of the TTU to the water is not enough to heat the water. Therefore, T_w is assumed as a constant.

The equation of convective heat transfer Φ_{conv} is as follows:

$$\Phi_{conv} = hA(T_{Co} - T_w) \quad \text{(S2)}$$

h is the convective heat transfer coefficient of the water.

According to the hydrodynamics formula of the water flows over the surface of the plate, h can be calculated by the follow equation [4]:

$$h_x/\lambda_w = 0.332 Re_x^{\frac{1}{2}} Pr^{\frac{1}{3}} \quad \text{(S3)}$$

Re_x is the Reynolds number of the water, and it can be calculated by the following equation:

$$Re_x = ux/v \quad \text{(S4)}$$

u is the water flow velocity, x is the distance of the water flowing over the plate, and v is the kinematic viscosity coefficient of the water.

P_r is the Prandtl number of the water, and it can be calculated by the following equation:

$$P_r = v/a \quad \text{(S5)}$$

a is the thermal diffusivity of the water.

$$v = \mu/\rho_w \quad \text{(S6)}$$

μ is the dynamic viscosity coefficient of the water, and ρ_w is the water density.

$$a = \lambda_w/\rho_w c_w \quad \text{(S7)}$$

c_w is the specific heat capacity of the water.

Integrating Equation (S3) over x:

$$hl/\lambda_w = 0.664 Re_l^{\frac{1}{2}} Pr^{\frac{1}{3}} \quad \text{(S8)}$$

l is the distance of the water flowing over the TTU.

$$h = 0.664 \lambda_w Re_l^{\frac{1}{2}} Pr^{\frac{1}{3}}/l \quad \text{(S9)}$$

Re_l is the Reynolds number of the water flowing the distance l.

Combining Equation (S2) and Equation (S8), Φ_{conv} can be calculated by the following equation:

$$\Phi_{conv} = 0.664\lambda_w Re_l^{\frac{1}{2}} Pr^{\frac{1}{3}} A(T_{Co} - T_w)/l \tag{S10}$$

As shown in Fig.S1(d)(II), in addition to Φ_{cond} and Φ_{conv}, there is also $\Phi_{cond\cdot C}$ exists. $\Phi_{cond\cdot C}$ is the heat transfers from the cold end of the TTU to the prototype shell and satisfies the following equation:

$$\Phi_{cond\cdot C} = \lambda_C A_C(T_{Co} - T_w)/D_C \tag{S11}$$

D_C is the thickness of the thermal conductivity layer between the TTU and the prototype shell, λ_C is the thermal conductivity of the prototype shell, and A_C is the contact area between the TTU and the prototype shell.

The total exothermic heat of the cold end of the TTU is $\Phi_{conv} + \Phi_{cond} + \Phi_{cond\cdot C}$. $\Phi_{conv} + \Phi_{cond} + \Phi_{cond\cdot C}$ satisfies the following equation:

$$\begin{aligned}
&\Phi_{conv} + \Phi_{cond} + \Phi_{cond\cdot C} \\
&\quad = 0.664\lambda_w Re_l^{\frac{1}{2}} Pr^{\frac{1}{3}} A(T_{Co} - T_w)/l + \lambda_w A(T_{Co} - T_w)/\delta D + \\
&\quad\quad \lambda_C A_C(T_{Co} - T_w)/D_C \\
&\quad = (T_{Co} - T_w)(0.664\lambda_w Re_l^{\frac{1}{2}} Pr^{\frac{1}{3}} A\delta D D_C)/\delta D l D_C + \\
&\quad\quad (T_{Co} - T_w)(\lambda_w A l D_C + \lambda_C A_C l \delta D)/\delta D l D_C
\end{aligned} \tag{S12}$$

Inside the TTU, there is conductive heat transfer $\Phi_{cond\cdot MTEG}$ from the hot end to the cold end, which can be calculated by the following equation:

$$\Phi_{cond\cdot MTEG} = \lambda_{MTEG} A(T_H - T_{Co})/D_{MTEG} \tag{S13}$$

T_H is the hot end temperature of the TTU, D_{MTEG} is the thickness of the thermal conductive layer which is between the cold end and hot end of TTU, and λ_{MTEG} is the thermal conductivity of the TTU.

The hot end of the TTU is connected to the heat source, and the heat source outputs thermal power P to the hot end of the TTU.

The total heat flow Φ_H of the hot end of the TTU can be calculated by the following equation:

$$\begin{aligned}
\Phi_H &= P - \Phi_{cond\cdot MTEG} \\
&= (D_{MTEG} P - \lambda_{MTEG} A(T_H - T_{Co}))/D_{MTEG}
\end{aligned} \tag{S14}$$

The total heat flow Φ_{Co} of the cold end of the TTU is:

$$\begin{aligned}
\Phi_{Co} &= \Phi_{cond\cdot MTEG} - (\Phi_{conv} + \Phi_{cond} + \Phi_{cond\cdot C}) \\
&= \lambda_{MTEG} A(T_H - T_{Co})/D_{MTEG} - \\
&\quad (T_{Co} - T_w)\left(0.664\lambda_w Re_l^{\frac{1}{2}} Pr^{\frac{1}{3}} A\delta D D_C\right)/\delta D l D_C - \\
&\quad (T_{Co} - T_w)(\lambda_w A l D_C + \lambda_C A_C l \delta D)/\delta D l D_C
\end{aligned} \tag{S15}$$

Combining Equation (S14), Equation (S15) and equation $\Delta T = \Phi/c_p$, ΔT_H, which is the temperature variation of the hot end of the TTU, and ΔT_{Co}, which is the temperature variation of the cold end of the TTU, can be calculated by the following equation:

$$\Delta T_H = (D_{MTEG}P - \lambda_{MTEG}A(T_H - T_{Co}))/D_{MTEG}c_p \quad \text{(S16)}$$

$$\Delta T_{Co} = \lambda_{MTEG}A(T_H - T_{Co})/D_{MTEG}c_p - (T_{Co} - T_w)\left(0.664\lambda_w Re_l^{\frac{1}{2}} Pr^{\frac{1}{3}} A\delta DD_C\right)\Big/\delta DlD_C c_p - (T_{Co} - T_w)(\lambda_w AlD_C + \lambda_C A_C l\delta D)/\delta DlD_C c_p \quad \text{(S17)}$$

c_p is the specific heat capacity of the TTU.

According to the Seebeck effect, the output voltage of the TTU can be calculated by the equation $V = S(T_H - T_{Co})$. S is the Seebeck coefficient of the MTEG which is inside the TTU. Therefore, the output voltage V is:

$$\begin{aligned} V &= S(T_H + \Delta T_H) - S(T_{Co} + \Delta T_{Co}) \\ &= S(T_H D_{MTEG}c_p + D_{MTEG}P - \lambda_{MTEG}A(T_H - T_{Co}))/D_{MTEG}c_p \\ &\quad - ST_{Co} - S\lambda_{MTEG}A(T_H - T_{Co})/D_{MTEG}c_p + \\ &\quad S\,(T_{Co} - T_w)\left(0.664\lambda_w Re_l^{\frac{1}{2}} Pr^{\frac{1}{3}} A\delta DD_C\right)\Big/\delta DlD_C c_p + \\ &\quad S\,(T_{Co} - T_w)(\lambda_w AlD_C + \lambda_C A_C l\delta D)/\delta DlD_C c_p \end{aligned} \quad \text{(S18)}$$

Set k_1 and k_2 as:

$$\begin{aligned} k_1 &= S(T_H D_{MTEG}c_p + D_{MTEG}P - \lambda_{MTEG}A(T_H - T_{Co}))/D_{MTEG}c_p \\ &\quad - ST_{Co} - S\lambda_{MTEG}A(T_H - T_{Co})/D_{MTEG}c_p + \\ &\quad S\,(T_{Co} - T_w)(\lambda_w AlD_C + \lambda_C A_C l\delta D)/\delta DlD_C c_p \end{aligned} \quad \text{(S19)}$$

$$k_2 = S\,(T_{Co} - T_w)\left(0.664\lambda_w l^{\frac{1}{2}} Pr^{\frac{1}{3}} A\delta DD_C\right)/\delta DlD_C c_p v^{\frac{1}{2}} \quad \text{(S20)}$$

The equations of k_1 and k_2 do not contain u, the u does not affect them. Therefore, the k_1 and k_2 are constants. Thus, the relationship equation between V and u satisfies the equation:

$$V = k_1 + k_2 u^{\frac{1}{2}} \quad \text{(S21)}$$

According to Equation (S21), the relationship between u and V satisfies the equation:

$$u = ((V - k_1)/k_2)^2 \quad \text{(S22)}$$

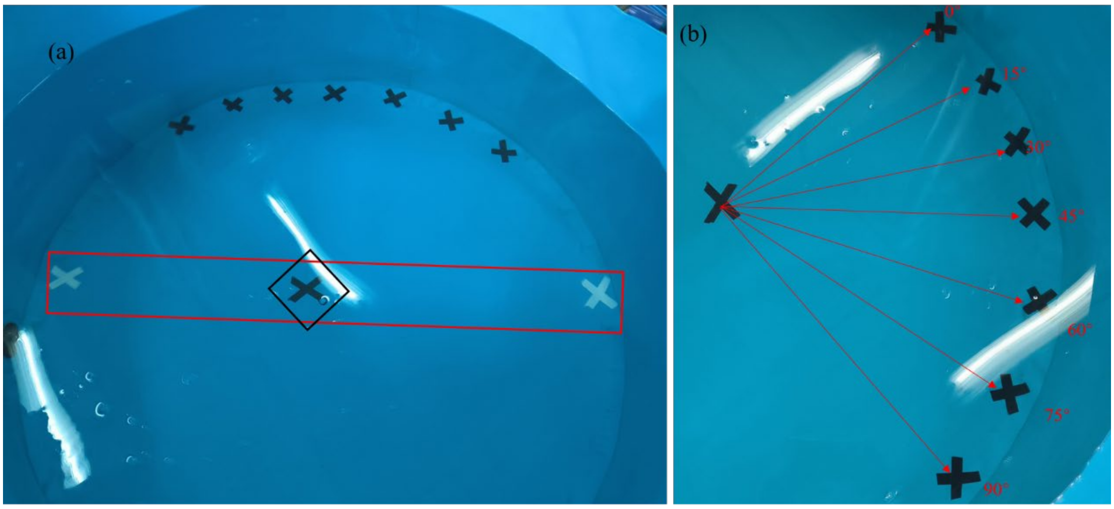

Figure S2. Water flow angle determination of experiment

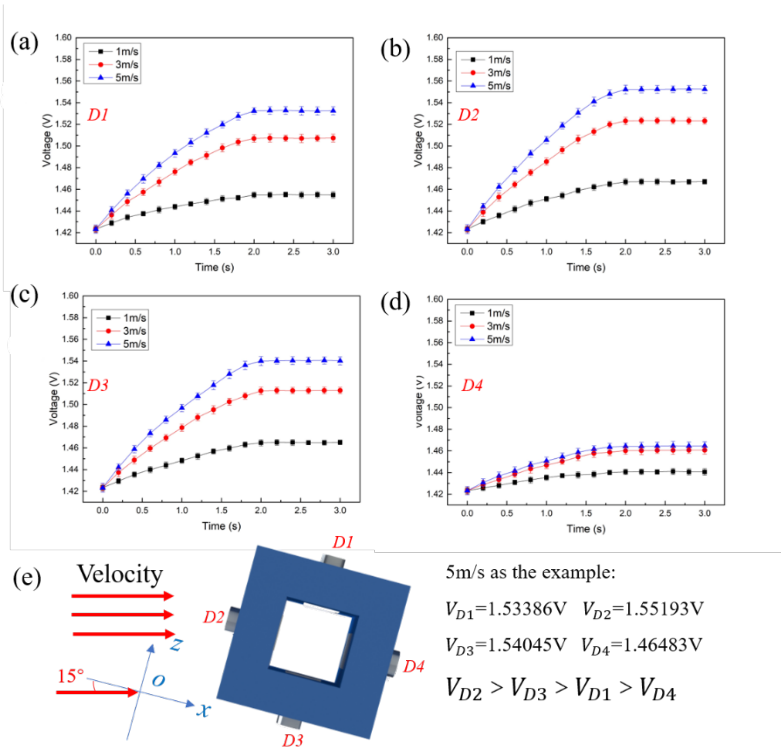

Figure S3. Output voltage of the prototype when the flow direction is 15° to the x-axis

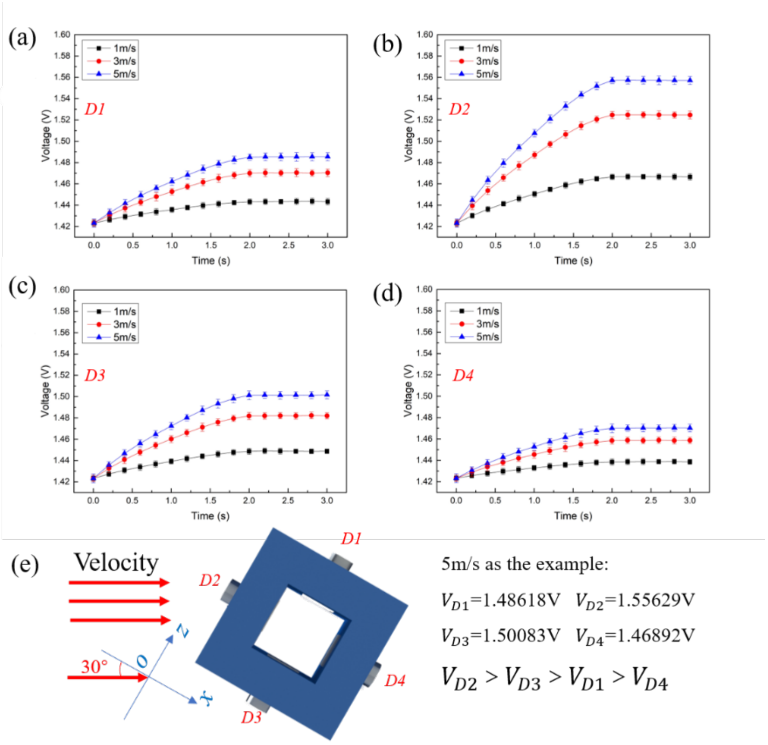

Figure S4. Output voltage of the prototype when the flow direction is 30° to the x-axis

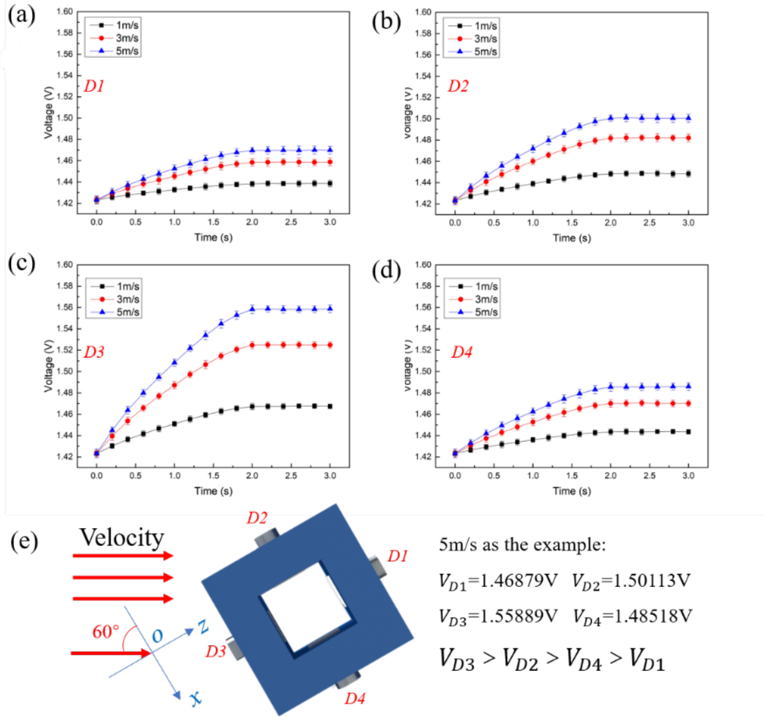

Figure S5. Output voltage of the prototype when the flow direction is 60° to the x-axis

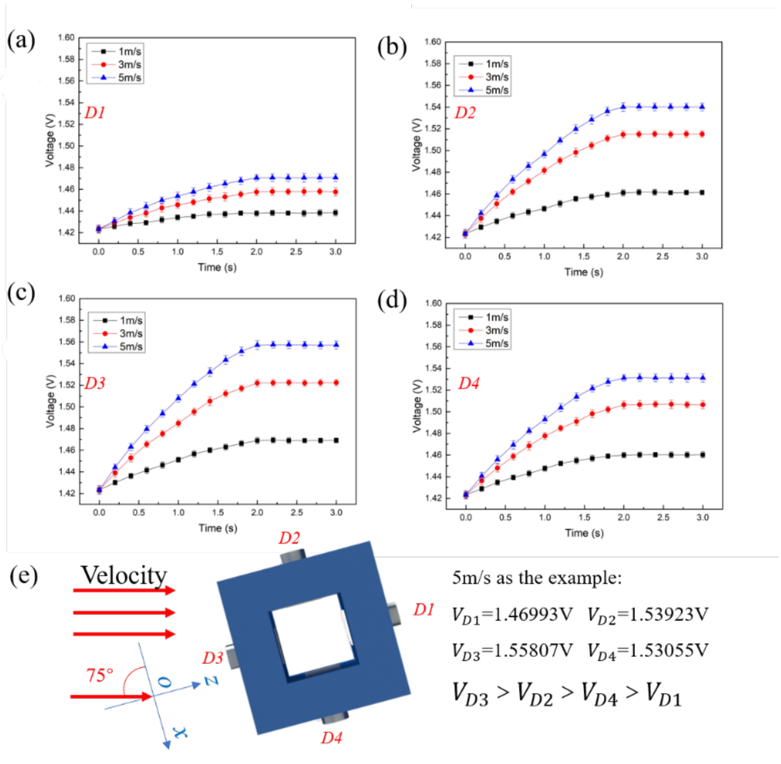

Figure S6. Output voltage of the prototype when the flow direction is 75° to the x-axis

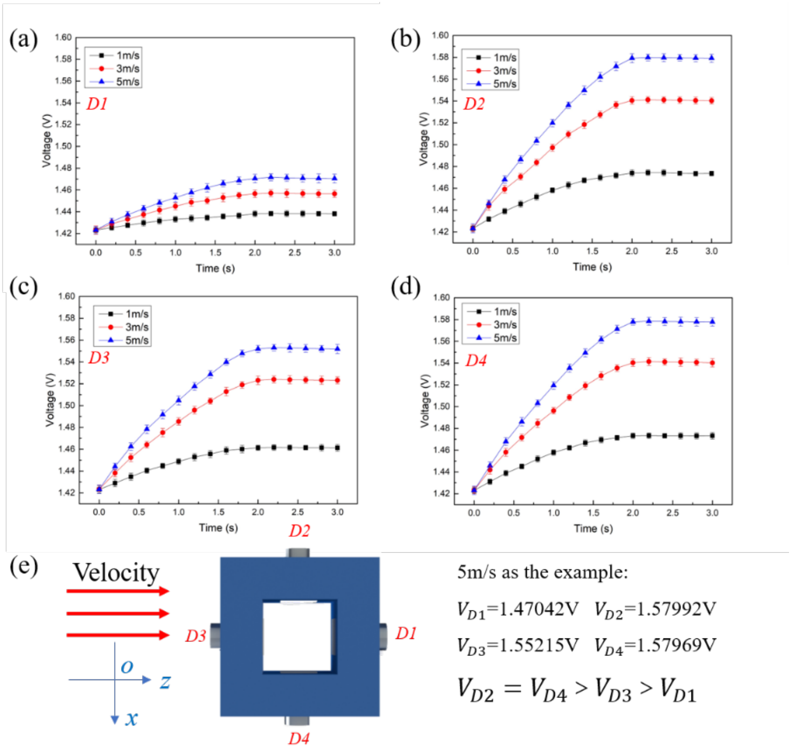

Figure S7. Output voltage of the prototype when the flow direction is 90° to the x-axis

Table S1. The performance comparisons between traditional sensors and thermal tactile sensor

Sensor type	Price	Accuracy	Dimension	Weight
Mechanical Current Meter	＞$ 3200	0~360°, ≤±4°	230×120×60 mm^3	3080 g
Electromagnetic Current Meter	＞$ 8700	0~360°, ≤±2°	216×120×146 mm^3	1650 g
Acoustic Current Meter	＞$ 2800	0~360°, ≤±5°	3.14×64×21.5 mm^3	4200 g
Thermal Tactile Underwater Flow Direction Sensor	＞$ 329	0~360°, ±15°	50×50×34 mm^3	200 g

Reference

[1] Guoneng Li, Zhihao Zhu, Youqu Zheng, Wenwen Guo, Yuanjun Tang. Development of a powerful hybrid micro thermoelectric generator based on an ultrahigh capacity miniature combustor. *Applied Thermal Engineering*, 2022, 206: 118039.

[2] ChangxinLiu*, BaichuanShan, NanxiChen, JianhaoLiu, ZhenghuiZhou, QingyongWang, YuGao, YunfeiGao, ZhitaoHan, ZhijianLiu, MinyiXu. A material recognition method underwater application based on Micro Thermoelectric Generator [J]. Sensors and Actuators:A. Physical, 2022.339(113503)

[3] Tao WQ. Heat Transfer (5th edition). 2019.

[4] Changxin Liu*, Guanghao Qu, and et.al. Underwater Hybrid Energy Harvesting based on TENG-MTEG for Self-powered Marine Mammal Condition Monitoring System, Materials Today Sustainability, DOI: https://doi.org/10.1016/j.mtsust.2022.100301

Appendix. Letter abbreviation and symbol table

**Letter abbreviation symbol**	**Representative meaning**	**Unit**
Φ_{cond}	Conductive heat transfer between the TTU and the water	W
Φ_{conv}	Convective heat transfer between the TTU and the water	W
T_{Co}	Cold end temperature of TTU	K
T_w	The water temperature	K
A	Contact area between the TTU and the water	m^2
δD	Thickness of the thermal conductive layer between the TTU and the water	m

λ_w	Conductive coefficient of the water	W/(m·K)
h	Convective heat transfer coefficient of the water	W/(m^2·K)
h_x	Convective heat transfer coefficient of the water with characteristic length x	W/(m^2·K)
Re_x	Reynolds number of the water with characteristic length x	/
u	The water flow velocity	m/s
x	Distance that the water flows	m
υ	Kinematic viscosity coefficient of the water	m^2/s
P_r	Prandtl number of the water	/
a	Thermal diffusivity of the water	m^2/s
μ	Dynamic viscosity coefficient of the water	(N·s)/m^2
ρ_w	Density of the water	kg/m^3
c_w	Specific heat capacity of the water	J/(kg·K)
l	Distance of the water flowing over the TTU	m
Re_l	Reynolds number of the water with characteristic length l	/
$\Phi_{cond\cdot C}$	Conductive heat transfer between the TTU and the sensor shell	W
D_C	Thickness of the thermal conductivity layer between the TTU and the sensor shell	m
λ_C	Thermal conductivity of the sensor shell	W/(m·K)
A_C	Contact area between the TTU and the sensor shell	m^2
$\Phi_{cond\cdot MTEG}$	Conductive heat transfer between the cold end and hot end of the TTU	W
T_H	Hot end temperature of the TTU	K
D_{MTEG}	Thickness of the thermal conductive layer between the cold end and hot end of TTU	m
λ_{MTEG}	Thermal conductivity of the TTU	W/(m·K)

Φ_H	Total heat flow of the hot end of the TTU	W
Φ_{Co}	Total heat flow of the cold end of the TTU	W
ΔT_H	Temperature variation of the hot end of the TTU	K
ΔT_{Co}	Temperature variation of the cold end of the TTU	K
c_p	Specific heat capacity of the TTU	J/(kg·K)
V	Output voltage of the TTU	V
S	Seebeck coefficient of the TTU	V/K
